# Women in surgery: do surgical specialties keep up with the feminization of medicine in Brazil?

**DOI:** 10.1590/0100-6991e-20233614-en

**Published:** 2023-11-10

**Authors:** Nyara Rodrigues Conde de Almeida, Lívia Guerreiro de Barros Bentes, Maria Fernanda de Almeida Cavalcante Aranha, Rafael Silva Lemos, Deivid Ramos dos Santos, Edson Yuzur Yasojima

**Affiliations:** 1 - Universidade do Estado do Pará, Laboratório de Cirurgia Experimental - Belém - PA - Brasil

**Keywords:** Medicine, Feminization, Women, Working, Physicians Distribution, Gender Equity, Medicina, Feminização, Mulheres Trabalhadoras, Distribuição de Médicos, Equidade de Gênero

## Abstract

**Introduction:**

historically, surgical medical specialties are mostly male, a scenario which, in recent years, has undergone changes. In this sense, despite the relevance of the growth of female participation in the medical career, little is discussed about the distribution between genders of the main surgical medical specialties in the country.

**Objective:**

discuss the process of feminization in surgical specialties in Brazil over the last few years, tracing a distribution profile of these specialties.

**Methods:**

this is a retrospective and cross-sectional study with secondary data from the Censuses of Medical Demography in Brazil in the years 2011, 2013, 2015, 2018, 2020 and 2023, including the surgical specialties: Urology, Orthopedics and Traumatology, Thoracic Surgery, Neurosurgery, Digestive System Surgery, Cardiovascular Surgery, Hand Surgery, General Surgery, Head and Neck Surgery, Vascular Surgery, Plastic Surgery, Ophthalmology, Coloproctology, Otorhinolaryngology, Pediatric Surgery, and Gynecology and Obstetrics.

**Results:**

males prevails in numbers, among the surgical specialties, however, with a lower growth rate compared to females. Specialties such as urology, orthopedics and traumatology and neurosurgery are mostly male, while gynecology and obstetrics are female.

**Conclusion:**

it is evident that female participation in the surgical medical field has increased significantly over the last few years.

## RESUMO

**Introdução:**

historicamente, as especialidades médicas cirúrgicas são, majoritariamente, masculinas, cenário o qual, nos últimos anos, passa por mudanças. Nesse sentido, apesar da relevância do crescimento da participação feminina na carreira médica, pouco se debate sobre a distribuição entre sexos das principais especialidades médicas cirúrgicas no país.

**Objetivo:**

discutir o processo de feminização nas especialidades cirúrgicas no Brasil ao longo dos últimos anos, traçando um perfil de distribuição dessas especialidades.

**Métodos:**

Trata-se de um estudo retrospectivo e transversal com dados secundários oriundos dos Censos de Demografia Médica no Brasil dos anos de 2011, 2013, 2015, 2018, 2020 e 2023, incluindo as especialidades cirúrgicas:Urologia, Ortopedia e Traumatologia, Cirurgia Torácica, Neurocirurgia, Cirurgia do Aparelho Digestivo, Cirurgia Cardiovascular, Cirurgia da Mão, Cirurgia Geral, Cirurgia de Cabeça e Pescoço, Cirurgia Vascular, Cirurgia Plástica, Oftalmologia, Coloproctologia, Otorrinolaringologia, Cirurgia Pediátrica e Ginecologia e Obstetrícia.

**Resultados:**

O sexo masculino prevalece em números, dentre as especialidades cirúrgicas, porém, com menor taxa de crescimento em comparação ao sexo feminino. Especialidades como a urologia, ortopedia e traumatologia e neurocirurgia são majoritariamente masculinas, enquanto a ginecologia e obstetrícia, feminina.

**Conclusão:**

evidencia-se que a participação feminina na área médica cirúrgica aumentou significativamente ao longo dos últimos anos.

## INTRODUCTION

In a global context, the growth of female participation in several areas previously dominated by men is evident^1^ ^,^ ^2^. Due to structural changes in society, the affirmation of wage employment, and the growing female predominance of the population, women were able to mitigate obstacles that were previously preventing them from having their full education, the same opportunities, and working hours^1^ ^-^ ^3^. This is called the feminization of the workforce, that is, a significant increase in women taking on positions previously held by men^4^.

In the last thirty years in European countries, the increase in the economically active population was due to the greater presence and participation of women. The same effect is seen in Latin America, where during the years 1960 to 1990, the number of economically active women tripled, growing from 18 to 57 million^5^. According to data from the 2021 Higher Education Census proposed by the Brazilian Institute of Geography and Statistics (IBGE)^6^, the profile of students entering Brazilian universities is mostly female.

A large part of this female workforce is concentrated in some sectors of activity, including domestic, administrative, teaching, and health. When it comes specifically to Brazilian medicine, despite men still comprising the majority of the active medical class, female participation has seen a slow and gradual increase over the last few decades, marked by the increase in the number of university graduates^2^ ^,^ ^4^ ^,^ ^7^ ^,^ ^9^. This disparity has decreased over the years, since men made up 51.4% of the Brazilian doctors in 2022, as opposed to 69.2% in the 90s. It is also estimated that women will be the majority in Brazilian medicine workforce by 2024^8^.

This change marks a process of feminization of medicine in the country, especially when analyzing younger age groups at the base of the doctors’ population pyramid^1^ ^,^ ^2^ ^,^ ^10^. However, specifically regarding surgical specialties, there is still a strong male predominance, as female general surgeons account for only 23.4% of professionals^8^. In subspecialties such as neurosurgery, orthopedics, and urology, women make up an even smaller number, 9.4%, 7.4%, and 2.9% of the contingent, respectively^1^ ^-^ ^3^ ^,^ ^8^. This configuration arises, especially, from the challenges of routine and workload in the surgical career, in addition to existing social factors and stigmas ^9^ ^-^ ^11^.

In this sense, despite the relevance of the topic on female participation in the medical career, no current research was found in the literature that debated the distribution between sexes of the main surgical medical specialties in the country. Therefore, the objective of this work is to discuss the feminization process in surgical specialties in Brazil over the last few years, outlining a distribution profile of these specialties.

## METHOD

We conducted a retrospective, cross-sectional study with secondary data, which includes evaluating sociodemographic characteristics of doctors in Brazil according to the number of professional records, gender, and distribution in medical and surgical specialties.

The secondary data used correspond to the Medical Demography Censuses in Brazil from the years 2011, 2013, 2015, 2018, 2020, and 2023.

We considered the number of specialists the most suitable variable for mapping population characteristics, such as sex and age, as it disregards secondary medical records, that is, those registered with more than one Regional Council of Medicine (CRM) for practicing in more than one Federation Unit. We obtained specialist data from the Medical Demography Censuses, by crossing information from medical specialty societies, the National Medical Residency Commission (CNRM) of the Ministry of Health, and the Regional Medical Councils, as there is no unified database in the country^8^. Those who do not have a degree recognized by one of these entities are not considered specialists.

We included the following medical specialties: Urology, Orthopedics and Traumatology, Thoracic Surgery, Neurosurgery, Digestive System Surgery, Cardiovascular Surgery, Hand Surgery, General Surgery, Head and Neck Surgery, Vascular Surgery, Plastic Surgery, Ophthalmology, Coloproctology, Otorhinolaryngology, Pediatric Surgery, and Gynecology and Obstetrics.

We present the data in raw numbers, percentages, and by the male-female ratio (MFR), which constitutes descriptive statistics, calculated from the number of males divided by the number of females in the same surgical specialty. Values less than 1 indicate a predominance of females in relation to males, while values greater than 1 indicate male predominance. In turn, a ratio equal to 1 indicates equality between the groups. We tabulated and organized the obtained data in Microsoft Office Word and Microsoft Office Excel, both in the 2016 version.

To demonstrate the temporal distribution of the percentage of male and female specialists, we created maps with pie charts using the QGIS 3.22 software. The country’s demographic and cartographic data were obtained from the Brazilian Institute of Geography and Statistics (IBGE), comprising data available in the Medical Demography Censuses in Brazil for the years 2011, 2013, 2015, 2018, 2020, and 2023.

Since we extracted the data from public domain documents, submission to the Ethics in Research Committee was not necessary.

## RESULTS

**Figure 1:**
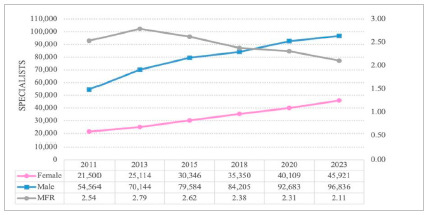
Number of specialist doctors according to sex and male-female ratio in Brazil, in the years 2011, 2013, 2015, 2018, 2020, and 2023.

Regarding the total number of specialist doctors, males remain with significant numbers, totaling, in 2023, 96,836 doctors, as opposed to 45,921 female physicians. At the same time, the growth rate in the number of female specialists increased by 114%, in contrast to the 77% growth for male doctors (Figure 1).

**Figure 2:**
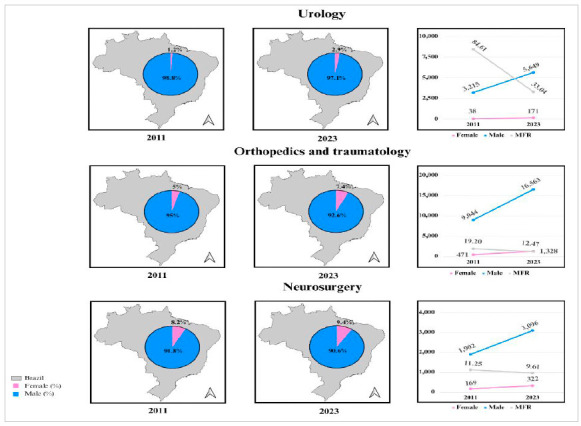
Percentage, absolute number, and male-female ratio of specialists in urology, orthopedics and traumatology, and neurosurgery in 2011 and 2023 in Brazil.

**Figure 3:**
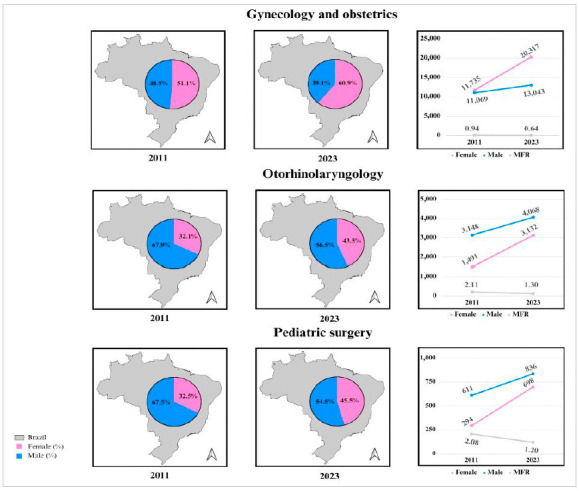
Percentage, absolute number, and male-female ratio of specialists in gynecology and obstetrics, otorhinolaryngology, and pediatric surgery in 2011 and 2023 in Brazil.

Certain specialties remain predominantly male, such as urology, followed by orthopedics and traumatology and neurosurgery (Table 1 and Figure 2). On the other hand, gynecology and obstetrics represents an inversion of the MFR, with female predominance in all years analyzed. Other specialties, such as otorhinolaryngology and pediatric surgery, show less discrimination between men and women (Table 1 and Figure 3).

**Table 1:** Distribution of surgical specialties according to sex and male-female ratio in Brazil, in the years 2011, 2013, 2015, 2018, 2020, and 2023.

SPECIALTIES	2023			2020			2018			2015			2013			2011		
	F	M	MFR	F	M	MFR	F	M	MFR	F	M	MFR	F	M	MFR	F	M	MFR
Urology	171	5,649	33.04	125	5,284	42.27	108	4,819	44.62	89	4,702	52.83	69	4,001	57.99	38	3,215	84.61
Orthopedics and Traumatology	1,328	16,563	12.47	1,045	14,954	14.31	916	13,213	14.42	775	12,372	15.96	540v	9,954	18.43	471	9,044	19.20
Neurosurgery	322	3,096	9.61	279	2,902	10.40	248	2,638	10.64	223	2,652	11.89	201	2,226	11.07	169	1,902	11.25
Thoracic surgery	134	941	7.02	104	893	8.59	85	811	9.54	74	839	11.34	54	709	13.13	32	459	14.34
Digestive System Surgery	417	2,943	7.06	323	2,655	8.22	274	2,382	8.69	210	2,142	10.20	152	1,832	12.05	91	964	10.59
Cardiovascular Surgery	249	1,975	7.93	228	1,962	8.61	215	1,847	8.59	205	2,015	9.83	189	1,806	9.56	110	992	9.02
Hand Surgery	158	822	5.20	140	710	5.07	118	614	5.20	77	508	6.60	46	364	7.91	27	175	6.48
Head and Neck Surgery	255	929	3.64	201	883	4.39	172	805	4.68	136	793	5.83	87	544	6.25	53	331	6.25
General surgery	8,286	27,165	3.28	7,605	26,874	3.53	6,447	24,321	3.77	4,452	19,741	4.43	3,661	18,599	5.08	2,206	11,400	5.16
Vascular surgery	1,370	3,591	2.62	1,112	3,362	3.02	916	3,022	3.30	706	2,835	4.02	511	2,370	4.64	331	1,543	4.66
Plastic surgery	1,645	4,895	2.98	1,470	4,682	3.19	1,294	4,249	3.28	1,178	4,453	3.78	989	3,823	3.87	799	3,213	4.02
Coloproctology	773	1,407	1.82	652	1,370	2.10	560	1,262	2.25	455	1,264	2.78	339	1,105	3.26	203	670	3.30
Ophthalmology	6,666	8,913	1.34	5,699	8,255	1.45	5,062	7,477	1.48	4,389	7,374	1.68	3,616	6,237	1.72	3,450	5,828	1.69
Otorhinolaryngology	3,132	4,068	1.30	2,701	3,972	1.47	2,311	3,658	1.58	1,951	3,752	1.92	1,610	3,364	2.09	1,491	3,148	2.11
Pediatric surgery	698	836	1.20	586	828	1.41	527	768	1.46	466	822	1.76	410	835	2.04	294	611	2.08
Gynecology and Obstetrics	20,317	13,043	0.64	17,839	13,097	0.73	16,097	12,319	0.77	14,960	13,320	0.89	12,640	12,375	0.98	11,735	11,069	0.94
TOTAL	45921	96836	2,11	40109	92683	2,31	35350	84205	2,38	30346	79584	2,62	25114	70144	2,79	21500	54564	2,54

F: female; M: male; MFR: male/female ratio

The biggest drop in the MFR was in the urology sector, falling from 84.61 (2011) to 33.04 (2023), followed by thoracic surgery, from 14.34 to 7.02, and orthopedics and traumatology, varying from 19.2 to 12.47 (Table 1).

## DISCUSSION

As occurred in other professional classes, Brazilian medicine is undergoing a change in the distribution between the sexes, the effect of a movement in favor of equal rights and a reduction in inequalities and discrimination in the work environment^2^ ^-^ ^5^. During the years of the study, there was an increase in female participation in surgical specialties, representing the beginning of an important process of feminization in Brazilian medicine. This process, however, proved to be slow and gradual over the decades^3^ ^-^ ^5^.

It was only at the end of the 19^th^ century that the first female medical students graduated in Brazil^15^ ^,^ ^16^. At the beginning of the 20^th^ century, men made up 77.7% of the medical profession. This male expressiveness lasted until the 60s, when it reached 87%. Following the opening of more medical schools and the significant fight for rights, female participation increased from 15.8% in 1970 to 48.6% in 2022^8^ ^,^ ^9^. The same occurs in the United States, given that, currently, women comprise 50% of those enrolled in medical universities^17^. When comparing the 2011 and 2023 demographic censuses, we also observed this increase in female representation in surgical specialties, resulting in a 114% growth rate in the number of female specialists^8^ ^,^ ^13^.

Despite the reduction in sex discrepancy over recent years, surgical specialties are still predominantly male. This same scenario is found in the United Kingdom and the United States, as men represent 73% and 61% of practicing surgeons, respectively. Among the surgical specialties studied, urology and orthopedics and traumatology were the specialties marked by the numerical superiority of men, despite the significant reduction in the discrepancy between sexes. Studies indicate that these surgical areas are, historically, demarcated by gender discrimination and psychological harassment, which, although often unrecognized, prevent women from entering them^3^ ^-^ ^5^ ^,^ ^15^ ^,^ ^16^.

When asked about the reasons, among other factors listed, the precarious maternity policy and work relations in educational institutions and hospitals, as well as the restricted guidance and mentoring on the part of teaching superiors and the salary difference are important causes of dissatisfaction and low adherence of women in surgical and academic careers^14^ ^,^ ^15^ ^,^ ^17^ ^-^ ^19^. These are characteristics still found in surgical areas such as urology, traumatology, and neurosurgery, which justify male supremacy in these areas, although there has been a decrease in MFR in recent decades^14^ ^-^ ^16^.

Another critical point strictly related to sex discrimination refers to female surgeons being repeatedly questioned about their professional and social skills, which is reflected in the limited number of women taking on leadership and coordination positions in surgical areas^16^ ^-^ ^21^. This condition is even more significant as one ascends the hierarchy of management positions^15^ ^,^ ^16^. Some studies demonstrate that, although mortality, complication, and readmission rates are similar regardless of the surgeon’s sex, when comparing productivity and team performance, female participation is associated with better surgical outcomes^21^. Others, in turn, point to lower rates of adverse postoperative outcomes in procedures performed by female surgeons compared with the ones carried out by male professionals^22^ ^,^ ^23^.

Conversely, surgical specialties focused on women’s health or that simultaneously have an associated clinical career, such as gynecology and obstetrics, otorhinolaryngology, and pediatric surgery, have a greater female participation and, therefore, display the lowest MFR rates. This is due to more valued and respected maternity policies, more flexible work routines and hours, in addition to more harmonious and balanced working relationships with superiors^14^ ^-^ ^16^ ^,^ ^18^ ^,^ ^20^ ^,^ ^21^ ^,^ ^24^.

In fact, when analyzing the years studied, there was a significant increase in women in surgical specialties, especially due to the fight for labor rights and the deconstruction of cultural paradigms and social stereotypes^15^ ^-^ ^17^ ^,^ ^24^ ^,^ ^25^. This condition is repeated in other countries, which noted that negative attitudes based on sex discrimination inhibit the career aspirations of female surgeons, posing a barrier to professional access and growth. Otherwise, encouraging better working conditions and environments and investing in professional career guidance and mentoring are factors that positively influence the entry of women into surgery^15^ ^-^ ^21^ ^,^ ^24^ ^,^ ^25^.

As limitations of the study, it is worth mentioning that a single data source - the Medical Demographic Census - can contribute to a smaller sample number of data and, therefore, mischaracterize the numerical reality of the proportion between men and women in surgical specialties in Brazil. Furthermore, it is not possible to know what is the main dedication or composition of the journey of doctors who have degrees in more than one specialty, which may not reflect reality.

## CONCLUSION

Female participation in the medical and surgical field has increased significantly over recent years, with a greater proportion of growth and participation in certain specialties. Although this great advance has been demonstrated, educational measures related to coexistence in the workplace and labor rights are still necessary, in search of a greater increase in the surgical training of women, aiming at equal participation between men and women in this field.
